# Regional Distribution of the Anthropometric Failure among Under-five Children and Its Determinants in India

**DOI:** 10.4314/ejhs.v33i3.11

**Published:** 2023-05

**Authors:** Ramendra Nath Kundu, Juri Borah, Susmita Bharati, Premananda Bharati

**Affiliations:** 1 Research Associate-I, Indian Council of Medical Research - Centre for Ageing & Mental Health, Kolkata, West Bengal, India; 2 Assistant Professor and Head, Department of Anthropology, Gurucharan College, Silchar, Assam; 3 Former Scientist, Sociological Research Unit, Indian Statistical Institute, Kolkata, West Bengal, India; 4 Former Professor and Head, Biological Anthropology Unit, Indian Statistical Institute, Kolkata, West Bengal, India

**Keywords:** Indian Regions, Anthropometric Failure, Child Undernutrition, Child Anemia, Public Health

## Abstract

**Background:**

Undernutrition in children seems to be one of the major health issues in developing nations including India. Stunting, underweight, and wasting are the three most often used anthropometric indicators to evaluate childhood undernutrition. Children who exhibit one or more indicators of undernutrition are considered as anthropometric failure (AF). The present study aims to determine the distribution and determinants of anthropometric failure in children under the age of five in different regions of India.

**Methods:**

NFHS-5 data, collected between 2019 and 2021, were utilized for the study. Pearson's chi-square (χ^2^) test was used to look into the association between categorical variables. Binary logistic regression was used to find the explanatory factors that influence anthropometric failure.

**Results:**

More than half of the under-five children (52.18%) in India are suffering from anthropometric failure, out of these West (57.88%), East (56.58%), and Central (53.94%) regions have covered half of the total occurrence. State-wise, Bihar (61.66%), followed by Gujarat (60.26%), and Jharkhand (58.05%) have recorded the highest rates of anthropometric failure. Anthropometric failure is higher among anemic children, boys, parent not alives, the higher number of birth order, lower educated mothers, rural dwellers, belonging to scheduled tribes and scheduled castes communities, living in nuclear families, and having lower household wealth indexes than their other counterparts.

**Conclusion:**

These aspects imply that regional determinants should be taken into consideration when implementing child nutrition development programs.

## Introduction

India is a developing nation with more than 119.84 million under-five children, or 9.90 percent of the total population according to of the 2011 census. By 2021, that number was projected to be over 114.27 million, constituting 8.38 percent of the total population (1). These children are the most significant assets that will shape the future nation. The health and nutritional status of newborns and children are used to determine the growth evaluation (2). Early nutrition is crucial for a child's healthy development, organ formation and function, strong immune system, neurological and cognitive progress (3).

Undernutrition in children seems to be one of the major health issues in a developing country. According to estimates, globally, undernutrition in various forms accounts for 45.0 percent of child fatalities each year (4). Childhood malnutrition affects the socioeconomic and medical well-being of individuals and households (5). According to UNICEF (2020), approximately half of all fatalities among children under the age of five are caused by undernutrition. Undernutrition in children has complex and multiple root causes (6). Previous research in several countries has found certain determinants of undernutrition, such as food insecurity, poor socio-economic situations, socio-demographic features, poverty, presence of comorbidities, inappropriate feeding habits, and insufficient complementary nutrients (7–15).

Anthropometry helps in evaluating childhood undernutrition. Stunting (low height-for-age), underweight (low weight-for-age), and wasting (low weight-for-height) are the three most often used anthropometric indicators that are universally accepted for undernutrition assessment. Poor nutrition during pregnancy and early childhood may have devastating effects like stunting and wasting. The key finding reports for the 2021 edition of the Joint Child Malnutrition Estimates mentioned that in 2020, globally, 149.2 million (22.0%) and 45.5 million (6.7%) children under five years were suffering from stunting and wasting, respectively (16).

Svedberg (2000) proposed an elaborate Composite Index of Anthropometric Failure (CIAF) for assessing malnutrition and noted that the conventional indices were insufficient to measure the overall prevalence of child undernutrition. According to Svedberg, children who exhibit stunting, wasting and underweight are all regarded as being undernourished or being in a state of anthropometric failure (7). Nandy et al. (2005) used the composite index of anthropometric failure on Indian data and suggested using it instead of the three traditional indicators (stunting, wasting, and underweight) of undernutrition (17). Since then, countries such as Ethiopia, Yemen, Bangladesh, Tanzania, and others adopted the composite index of anthropometric failure model to determine child undernutrition status (10–12, 18–19).

Using the composite index of anthropometric failure, a few studies have evaluated the prevalence of undernutrition in Indian children. Most of the studies are found to be conducted on community-based and geography-specific. A few of these studies have also examined confounding factors regionally, like child age and sex, socioeconomic status, maternal education, birth order, birth intervals, exclusive breastfeeding, childhood morbidities, and the number of siblings. In this regard, a mention may be made of some of the studies conducted in India.

Shit and others studied among slum children in the Bankura district of West Bengal (20); Boregowda and others studied among urban slums of Raipur City of Chhattisgarh (21); Dhok and Thakre studied among slum children in Nagpur city (22); Kherde, and others studied among children attending the Immunoprophylaxis clinic in a tertiary care hospital in Nagpur (23), Kramsapi and others studied among preschool tribal children of Assam (24), Roy and others studied among the rural area of West Bengal (25), Basu and others studied among school children in North 24 Parganas district of West Bengal (26), and Vakilna and Nambiar studied among children of urban Surat in Western Gujarat (27).

The present study aims to estimate the distribution of anthropometric failure among under-five children and its determinants across the regions in India.

## Methods

**Figure 1:**
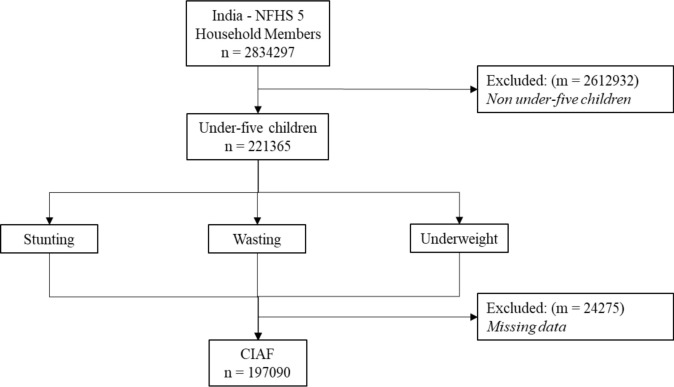
Sample determination for the analysis of anthropometric failure in India

**Data source**: The data was derived from the Fifth National Family Health Survey (NFHS-5), which was carried out between 2019 and 2021 in India. Anthropometric failure among children under the age of five was studied using the NFHS-5 data. Out of the 2834297 reported household members, 197,090 children under the age of five were included in the study. The criteria for data inclusion and exclusion were provided in the flow chart below (Figure 1). The complete data on stunting, wasting, and underweight were included in the valid sample. All invalid samples, including those with several missing or incomplete anthropometric measurements, were excluded from the study.

**Outcome variables- Anthropometric failure**: An indicator of child undernutrition is anthropometric failure (AF), which was used as the outcome variable and was derived from the composite index of anthropometric failure (CIAF). The anthropometric failure indicates insufficient and poor nutrition, which was determined by using nutritional indicators of stunting, wasting, and underweight (Table 1) (20). Stunting, wasting, and underweight were identified using the z-score. According to WHO, a z-score of less than -2.0 SD in height-for-age was determined as stunting, weight-for-height as wasting, and weight-for-age as underweight. The CIAF was categorized as binary mode in the data analysis, namely Anthropometric failure and No-failure (28, 29). Anthropometric failure (AF) was defined as children with stunting, wasting, and underweight, which indicated insufficient height and weight for their age (AF = ∑ Group B, C, D, E, F, Y), whereas no-failure was defined as children with no anthropometric failure (NF = Group A), which is shown in Table 1.

**Table 1:** Classification of the composite index of anthropometric failure among under-five children

Groups	Description	Wasting	Stunting	Underweight
A	No failure	No	No	No
B	Wasting only	Yes	No	No
C	Wasting and Underweight	Yes	No	Yes
D	Wasting, Stunting, and Underweight	Yes	Yes	Yes
E	Stunting and Underweight	No	Yes	Yes
F	Stunting only	No	Yes	No
Y	Underweight only	No	No	Yes

Note: No failure (NF) = A; Anthropometric failure (AF) = ∑ B, C, D, E, F, Y

**Explanatory variables - child, maternal, and socio-demographic determinants**: Studies on potential determinants of anthropometric failure in children focused on child health and demographics, maternal factors, and socio-demographic factors. Explanatory factors were explicitly classified while retaining their meaning by utilizing information from existing studies.

Anemia in children (anemic, non-anemic), gender of the children (boy, girl), and parents alive (alive, not alive) have been shown as child health-and-demographic variables (30, 31). Birth order (1st & 2nd, 3rd & more) and mother's education (non-educated, primary, secondary, higher) have been shown as maternal factors (32, 33). In this context, maternal education referred to the mother's formal educational background. Social category (ST, SC, OBC, general), family type (nuclear, non-nuclear), and wealth index (poor, middle, rich) have been shown as socio-demographic variable (34–36). The acronyms ST, SC, and OBC were used to designate scheduled tribes, scheduled castes, and other backward classes, respectively.

**Regional divisions in India**: India consists of 28 States and 8 Union Territories (UTs), which were grouped into 6 geographic regions by NFHS-5: Central, East, North, Northeast, South, and West (37). The Central region encompasses the states of Uttar Pradesh, Madhya Pradesh, and Chhattisgarh. The Eastern region is comprised of the states of Bihar, Jharkhand, Odisha, and West Bengal. The North region is comprised of states namely, Haryana, Himachal Pradesh, Punjab, and Rajasthan, and UTs namely Chandigarh, Jammu and Kashmir, and Ladakh with the capital Delhi. The region includes the Tibetan Plateau, the Himalayas, and a dessert known as “Thar”. Arunachal Pradesh, Assam, Manipur, Meghalaya, Mizoram, Nagaland, Sikkim, and Tripura are the eight states that constitute the Northeast region. The states of Andhra Pradesh, Karnataka, Kerala, Tamil Nadu, and Telangana, as well as the UTs of the Andaman and Nicobar Islands, Lakshadweep, and Puducherry, constitute the Southern region. The states of Goa, Gujarat, and Maharashtra as well as the UTs of Dadra & Nagar Haveli and Daman & Diu are all a part of the West region.

**Statistical analysis**: Frequency and percentage distribution for discrete variables were included in the descriptive analysis. Pearson's chi-square (χ^2^) test was used to look at the association between categorical variables. Binary logistic regression was used to find the explanatory factors that influence AF, where AF was coded as “1” and its counterpart, NF, as “0”. The independent variables were chosen following a multicollinearity test and a VIF of less than 5 was selected for analysis. Using a 95% confidence interval and a p-value of ≤0.05, statistical significance was determined. The Omnibus chi-square was used to confirm whether the data fit the logistic regression model (39, 40). Statistical Package for the Social Sciences (SPSS, version 25.0) and Microsoft Excel were used for all statistical analyses.

**Ethical approval**: The Demographic and Health Surveys (DHS) provided the NFHS-5 data that were used in this study. The survey protocols and participant confidentiality have been evaluated and approved by the ICF Institutional Review Board (IRB) and the International Institute for Population Sciences (IIPS) in India, a nodal organization of the host country. The ICF IRB adheres to the guidelines established by the US Department of Health and Human Services concerning participant confidentiality and the protection of human subjects. As a result, the DHS data did not require further ethical approval because it was ethically appropriate.

## Results

**Figure 2:**
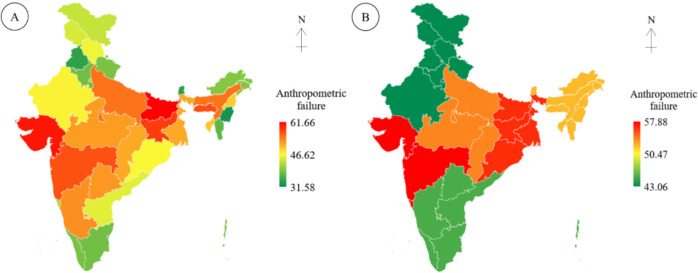
Prevalence of anthropometric failure among under-five children in India, (A) State wise distribution and (B) Region wise distribution.

**Figure 3:**
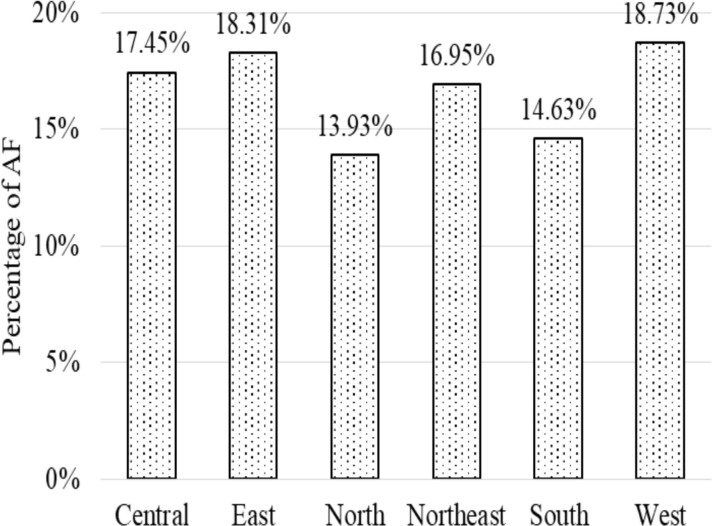
Percentage sharing of anthropometric failure across the region.

The prevalence of anthropometric failure among children under the age of five in the Indian populations on state-wise and regional basis are displayed through the maps in Figure 2. The findings reveal that one in two Indian children (52.18%) suffer from anthropometric failure (AF). State-wise, Bihar (61.66%), followed by Gujarat (60.26%), and Jharkhand (58.05%) have the highest AF rates, while Puducherry (31.58%), Chandigarh (32.38%), and Manipur (32.77%) has the lowest. In terms of regional distribution, the West (57.88%), and the East (56.58%) have the highest prevalence, while the North (43.06%) and South (45.23%) have the lowest prevalence. However, half of the total number of AF children in the country is recorded from West, East, and Central India (Figure 3).

In India, it is found that the group E (stunting and underweight) and group F (stunting only) predominately affect about 30.66 percent of children, while the group Y (underweight only) and group D (wasting, stunting, and underweight) comprises only 7.51 percent of children (Table 2). Maximum AF on the regional basis is recorded in the West (57.88%), followed by the East (56.58%) and the Central (53.94%). Children with all forms of undernutrition (group D) are more common in the West (7.48%) and East (6.40%) than in the rest of the country. Children from the Central (17.33%), Northeast (17.13%), North (14.69%), and South (13.40%) are more affected by only stunting (group F), whereas children from the East (18.10%) and West (15.85%) are more affected by both stunting and underweight (group E). West has the highest incidence of only wasting (group B) and only underweight (group Y) among all the regions, at 8.05 percent and 2.81 percent, respectively. A significant (p <0.001) difference is observed across the regions in the CIAF group frequencies. In India, stunting and being underweight among children have become widespread public concerns due to the prevalence of groups E and F.

**Table 2:** Regional difference in the composite index of anthropometric failure of children under the age of five

Groups	Nutritional condition		Regional divisions					
	
		India % (n)	Central % (n)	East % (n)	North % (n)	Northeast % (n)	South % (n)	West % (n)
A	No failure	47.83 (94249)	46.06 (24649)	43.42 (22906)	56.95 (15157)	47.62 (3547)	54.76 (17739)	42.12 (10251)
B	Wasting only	6.45 (12721)	5.90 (3159)	6.52 (3442)	5.88 (1565)	7.45 (555)	6.30 (2040)	8.05 (1960)
C	Wasting and Underweight	7.55 (14887)	6.98 (3735)	8.68 (4580)	5.64 (1501)	7.38 (550)	6.57 (2128)	9.83 (2393)
D	Wasting, Stunting, Underweight	5.23 (10306)	4.87 (2608)	6.40 (3375)	3.09 (823)	4.47 (333)	4.16 (1346)	7.48 (1821)
E	Stunting and Underweight	15.54 (30637)	16.72 (8951)	18.10 (9553)	12.01 (3198)	14.00 (1043)	12.46 (4035)	15.85 (3857)
F	Stunting only	15.12 (29793)	17.33 (9279)	14.43 (7617)	14.69 (3910)	17.13 (1276)	13.40 (4339)	13.86 (3372)
Y	Underweight only	2.28 (4497)	2.14 (1148)	2.45 (1295)	1.74 (464)	1.95 (145)	2.35 (762)	2.81 (683)
			*Chi-Square, 3579.43 (p<0.001)					
	Anthropometric failure (∑ B to Y)	52.17 (102841)	53.94 (28880)	56.58 (29862)	43.05 (11461)	52.38 (3902)	45.24 (14650)	57.88 (14086)

* Chi-square among the regions

**Region-wise factor distribution:** Table 3 shows that throughout the regional divisions, the frequency of explanatory variables changes significantly (χ2, p <0.01), based on AF. The prevalence of AF in anemic children varies significantly by regional division, with west India accounting for around three out of every five (60.41%). Compared to girls, boys are found to have a higher rate of AF. Children whose parents were deceased have greater rates of AF, although only the central region shows a statistically significant difference. The anthropometric failure (AF) is much more common in the third or more children in comparison to the first and second children in each region. However, compared to the women with higher education, AF is noticeably higher among mothers with lower education in each region. In this case, maternal education exclusively refers to formal institutional education. The anthropometric failure is significantly more common among rural children than in urban ones. Children in Scheduled Tribe and Scheduled Caste communities are found to be more affected by AF than general caste children. The prevalence of AF in children from nuclear families is higher than that of non-nuclear families. Children from lower household wealth indexes or the poor and middle classes have a greater AF rate across the country.

**Table 3:** Distribution of anthropometric failure children according to explanatory factors and its distribution by regions

Explanatory Factors		Regional parts of India					
	
	India % (n)	Central % (n)	East % (n)	North % (n)	Northeast % (n)	South % (n)	West % (n)
Anemia in children							
Anemic	55.34 (65098)	56.63 (17747)	60.25 (19541)	44.52 (7206)	55.79 (2366)	49.11 (8536)	60.41 (9701)
Non-anemic	46.30 (25662)	49.91 (7202)	50.37 (7289)	37.65 (2610)	47.17 (1133)	39.24 (4441)	50.90 (2988)
χ^2^ (p-value)	1233.34 (<0.001)	180.19 (<0.001)	399.33 (<0.001)	93.79 (<0.001)	45.70 (<0.001)	269.71 (<0.001)	159.31 (<0.001)
Childs' sex							
Boys	52.98 (53880)	54.80 (14990)	56.56 (15478)	44.93 (6272)	53.78 (2004)	46.02 (7738)	59.26 (7397)
Girls	51.33 (48958)	53.07 (13889)	56.63 (14384)	40.99 (5189)	50.97 (1897)	44.38 (6912)	56.42 (6688)
χ^2^ (p-value)	53.67 (<0.001)	16.19 (<0.001)	0.02 (0.878)	42.19 (<0.001)	5.92 (0.015)	8.71 (0.003)	20.07 (<0.001)
Parents alive							
Not alive	55.51 (1008)	62.24 (333)	58.32 (270)	45.41 (94)	54.76 (46)	46.67 (147)	55.92 (118)
Both alive	52.15 (101781)	53.86 (28534)	56.57 (29576)	43.04 (11365)	52.34 (3850)	45.21 (14489)	57.91 (13966)
χ^2^ (p-value)	8.15 (0.004)	14.97 (<0.001)	0.57 (0.451)	0.47 (0.492)	0.20 (0.658)	0.27 (0.605)	0.34 (0.561)
Birth order number							
1st & 2nd	49.53 (70206)	51.30 (17849)	53.31 (19070)	40.91 (8090)	50.08 (2705)	43.93 (11992)	56.14 (10501)
3rd & more	59.31 (31186)	58.87 (10586)	64.24 (10383)	49.39 (3178)	58.49 (1151)	52.70 (2520)	64.10 (3366)
χ^2^ (p-value)	1468.64 (<0.001)	273.02 (<0.001)	541.40 (<0.001)	142.63 (<0.001)	40.82 (<0.001)	126.12 (<0.001)	106.62 (<0.001)
Mother's Education							
No education	63.44 (26104)	63.02 (8731)	68.28 (10483)	50.74 (2827)	60.31 (667)	61.79 (1530)	67.01 (1867)
Primary	58.45 (14170)	58.90 (4369)	60.38 (4427)	49.56 (1810)	60.69 (752)	54.44 (1116)	66.43 (1696)
Secondary	50.17 (50777)	51.70 (12640)	51.34 (13178)	42.03 (5269)	50.24 (2304)	45.93 (8676)	57.74 (8710)
Higher	38.64 (11771)	40.21 (3138)	39.97 (1760)	32.02 (1555)	34.30 (177)	37.09 (3328)	46.32 (1812)
χ^2^ (p-value)	4877.00 (<0.001)	1175.02 (<0.001)	1679.84 (<0.001)	443.61 (<0.001)	138.22 (<0.001)	587.91 (<0.001)	386.51 (<0.001)
Social category							
ST	59.58 (12115)	59.05 (2847)	62.73 (3379)	49.59 (1097)	49.66 (1026)	56.53 (1113)	68.40 (2654)
SC	55.95 (26269)	58.85 (8014)	61.08 (8221)	46.46 (3521)	53.71 (441)	49.19 (3800)	60.55 (2272)
OBC	51.37 (42934)	53.48 (14137)	56.57 (11268)	44.29 (3942)	47.46 (634)	43.46 (8069)	58.05 (4882)
General	44.34 (15643)	44.65 (3572)	47.96 (4417)	36.06 (2579)	50.25 (496)	38.75 (1274)	49.74 (3306)
χ^2^ (p-value)	1603.85 (<0.001)	463.16 (<0.001)	471.56 (<0.001)	222.29 (<0.001)	8.06 (0.045)	229.60 (<0.001)	367.51 (<0.001)
Family type							
Nuclear	55.62 (41249)	58.38 (11577)	60.13 (13666)	47.07 (3809)	55.08 (2076)	45.38 (5599)	61.10 (4520)
Non-nuclear	50.10 (61590)	51.35 (17302)	53.91 (16196)	41.30 (7651)	49.59 (1825)	45.14 (9051)	56.47 (9564)
χ^2^ (p-value)	565.04 (<0.001)	248.72 (<0.001)	203.36 (<0.001)	76.34 (<0.001)	22.49 (<0.001)	0.17 (0.676)	45.20 (<0.001)
Wealth index							
Poor	60.65 (55424)	60.39 (17258)	61.82 (23128)	52.34 (3472)	56.91 (3089)	57.75 (3678)	68.89 (4799)
Middle	50.48 (19692)	51.90 (4997)	48.51 (3963)	48.28 (2488)	44.38 (517)	47.55 (4475)	59.30 (3251)
Rich	41.57 (27723)	43.23 (6623)	38.54 (2771)	37.09 (5501)	34.50 (295)	39.12 (6498)	50.77 (6035)
χ^2^ (p-value)	5981.91 (<0.001)	1201.80 (<0.001)	1587.51 (<0.001)	506.22 (<0.001)	184.08 (<0.001)	673.59 (<0.001)	597.85 (<0.001)

**Effect of the explanatory factor on anthropometric failure (AF)**: Binary logistic regression is used in Table 4 to show the impact of explanatory factors on anthropometric failure in Indian children under the age of five. Omnibus Chi-Square for India (χ^2^ 8191.90), Central (χ^2^ 1838.76), East (χ^2^ 2529.44), North (χ^2^ 798.80), Northeast (χ^2^ 209.52), South (χ^2^ 1146.42), and West (χ^2^ 960.85) all have a significant level at p<0.001. These facts show that the models for India and each regional division fit the data well. Each regression model is significant (p <0.01), with the correct percentage of prediction for India, Central, East, North, Northeast, South, and West, being 59.50 percent, 59.13 percent, 62.28 percent, 59.40 percent, 58.35 percent, 59.24 percent, and 60.87 percent, respectively. The model reveals the following facts.

**Table 4:** Effect of explanatory factors on anthropometric failure among under-five children in India

			Regional parts of India											
			
Explanatory Factors	India		Central		East		North		Northeast		South		West	
						
	AOR (95% CI)	p-value	AOR (95% CI)	p-value	AOR (95% CI)	p-value	AOR (95% CI)	p-value	AOR (95% CI)	p-value	AOR (95% CI)	p-value	AOR (95% CI)	p-value
Anemia in children (Ref. Non-anemic)														
Anemic	1.35 (1.32, 1.38)	<0.001	1.28 (1.23, 1.33)	<0.001	1.37 (1.31, 1.43)	<0.001	1.27 (1.20, 1.35)	<0.001	1.31 (1.16, 1.48)	<0.001	1.37 (1.30, 1.44)	<0.001	1.35 (1.27, 1.44)	<0.001
Sex child (Ref. Girl)														
Boy	1.06 (1.04, 1.08)	<0.001	1.07 (1.03, 1.12)	<0.001	0.99 (0.95, 1.03)	0.534	1.15 (1.09, 1.22)	<0.001	1.12 (0.99, 1.26)	0.067	1.06 (1.01, 1.11)	0.030	1.08 (1.02, 1.15)	0.006
Parents alive (Ref. Alive)														
Not alive	1.02 (0.92, 1.13)	0.667	1.36 (1.12, 1.65)	0.002	0.91 (0.74, 1.13)	0.412	0.93 (0.68, 1.26)	0.628	1.16 (0.68, 1.99)	0.587	0.94 (0.74, 1.20)	0.634	0.78 (0.58, 1.04)	0.091
Birth order number (Ref. 1st & 2nd)														
3rd & more	1.17 (1.14, 1.20)	<0.001	1.11 (1.06, 1.16)	<0.001	1.22 (1.16, 1.28)	<0.001	1.13 (1.05, 1.20)	<0.001	1.15 (0.99, 1.33)	0.075	1.20 (1.12, 1.29)	<0.001	1.11 (1.03, 1.20)	0.005
Mother's Education (Ref. Higher)														
No education	1.80 (1.73, 1.87)	<0.001	1.79 (1.67, 1.92)	<0.001	2.08 (1.90, 2.27)	<0.001	1.50 (1.35, 1.66)	<0.001	1.97 (1.45, 2.68)	<0.001	1.81 (1.62, 2.02)	<0.001	1.46 (1.28, 1.65)	<0.001
Primary	1.55 (1.49, 1.62)	<0.001	1.57 (1.46, 1.70)	<0.001	1.51 (1.38, 1.67)	<0.001	1.56 (1.40, 1.74)	<0.001	2.14 (1.59, 2.89)	<0.001	1.50 (1.34, 1.67)	<0.001	1.64 (1.45, 1.86)	<0.001
Secondary	1.31 (1.27, 1.35)	<0.001	1.31 (1.23, 1.39)	<0.001	1.25 (1.16, 1.35)	<0.001	1.36 (1.25, 1.48)	<0.001	1.42 (1.10, 1.83)	0.007	1.27 (1.19, 1.35)	<0.001	1.32 (1.21, 1.43)	<0.001
Social category (Ref. General)														
ST	1.31 (1.26, 1.36)	<0.001	1.29 (1.19, 1.40)	<0.001	1.23 (1.13, 1.32)	<0.001	1.21 (1.08, 1.35)	0.001	0.92 (0.78, 1.09)	0.354	1.46 (1.29, 1.66)	<0.001	1.66 (1.51, 1.83)	<0.001
SC	1.27 (1.24, 1.32)	<0.001	1.40 (1.32, 1.50)	<0.001	1.30 (1.22, 1.38)	<0.001	1.27 (1.18, 1.37)	<0.001	1.07 (0.87, 1.31)	0.520	1.24 (1.13, 1.36)	<0.001	1.38 (1.27, 1.51)	<0.001
OBC	1.18 (1.15, 1.22)	<0.001	1.24 (1.17, 1.31)	<0.001	1.20 (1.13, 1.26)	<0.001	1.20 (1.11, 1.29)	<0.001	0.85 (0.71, 1.02)	0.078	1.13 (1.04, 1.23)	0.005	1.32 (1.23, 1.42)	<0.001
Family type (Ref. Non-nuclear)														
Nuclear	1.04 (1.02, 1.07)	<0.001	1.09 (1.05, 1.14)	<0.001	1.03 (0.99, 1.08)	0.144	1.13 (1.06, 1.20)	<0.001	1.08 (0.95, 1.22)	0.240	0.93 (0.89, 0.98)	0.006	1.11 (1.04, 1.19)	0.001
Wealth index (Ref. Rich)														
Poor	1.71 (1.67, 1.76)	<0.001	1.56 (1.48, 1.64)	<0.001	1.85 (1.73, 1.98)	<0.001	1.56 (1.44, 1.68)	<0.001	1.84 (1.50, 2.26)	<0.001	1.72 (1.61, 1.85)	<0.001	1.70 (1.57, 1.84)	<0.001
Middle	1.27 (1.24, 1.31)	<0.001	1.25 (1.18, 1.32)	<0.001	1.30 (1.20, 1.40)	<0.001	1.37 (1.27, 1.47)	<0.001	1.25 (0.99, 1.57)	0.062	1.28 (1.20, 1.35)	<0.001	1.26 (1.17, 1.36)	<0.001

All regions have shared a common predictor, child anemia, but the effect of anemia on AF is strongest in East and South regions, where it is 1.37 times more common than in children who are not anemic, followed by the West at 1.35 times and the Northeast at 1.31 times more prevalent. Boys are more likely than girls to have AF, with prevalence rates in the North and West regions being 1.15 and 1.08 times higher, respectively. Only in the Central region the children who lost their parents are 1.36 times more likely to have AF than their counterparts. Birth order has a maximum significant impact in the East and South, where it is 1.22 times and 1.20 times more prevalent in the third and more children compared to the first and second. Lower maternal education is found to be a common factor and to be positively associated with AF, with lower or non-educated mothers have a higher probability of having an AF child than a higher educated mothers.

Except for the Northeast, the social category is shown to be a prevalent factor across all regions. Children from the Scheduled Caste community are more likely than children from general castes suffer from AF in the Central, East, and North, but Scheduled Tribe children are more affected in the South and West regions. Children from nuclear families were more likely to suffer from AF in the Central, North, and West regions, but in the South, it was 7 percent more prevalent in the non-nuclear family (AOR: 0.93, CI: 0.89, 0.98). The prevalence of AF found to be higher in children from poor- and middle-wealth-indexed families than from wealthier ones. In the East, there is a maximum prevalence of AF found among poor families-about 1.85 times(AOR: 1.85, CI: 1.73, 1.98).

## Discussion

More than half of the under-five children (52.18%) in India are suffering from AF, out of which West (57.88%), East (56.58%), and Central (53.94%) regions have covered half of the total occurrences. State-wise, Bihar (61.66%), followed by Gujarat (60.26%), and Jharkhand (58.05%) have recorded the highest rates of AF and it is the West (57.88%), and the East (56.59%) areas in terms of regional basis. The prevalence of AF in India shows consistency with the Ethiopian administrative zones (53.78%) (12). However, the rate of AF is found to be more than that of Bangladesh (48.30%) (18), Tanzania (38.20%) (11), and less than it was recorded in rural Yemen (70.10%) (10).

In the case of CIAF, group E (stunting and underweight) and group F (stunting only) are found to predominately affect more than 30.0 percent of children, while group Y (underweight only) and group D (wasting, stunting, and underweight) comprise only 7.51 percent. Children in group E (stunting and underweight), group B (only wasting), group D (wasting, stunting and underweight), and group Y (only underweight) are more common in the West region, at 15.85 percent, 8.05 percent, 7.48 percent, and 2.81 percent respectively. The East region is mostly affected by group D (wasting, stunting, and underweight), and group E (stunting and underweight), the percentage is 6.40 percent and 18.10 percent, respectively. Children from the Central (17.33%), Northeast (17.13%), North (14.69%), and South (13.40%) are more were affected by group F (only stunting). The regional differences in CIAF may be due to climatic variation in geographical, political, and socio-cultural norms, and due to dietary-related factors in different zones (40). The similarity in the findings of having more occurrences of group E (18%), followed by group F (13%) than other groups are also reported by Islam and Biswas in 2020 (18). The higher contribution of the E category of CIAF (19.6%) to the aggregate CIAF was also mentioned by Al-Sadeeq and others in 2019 (10).

The rate of AF is found to be higher among anemic children, boys, deceased parents, the higher number of birth order, lower educated mothers, those living in rural areas, belonging to Scheduled Tribe and Scheduled Caste communities, those living in nuclear families, and belonging to families having lower household wealth indexes for the poor and middle classes than their other counterparts. The study by Islam and Biswas (2020) in Bangladesh also recorded the higher odds of AF among the mothers having a higher order of birth, living in rural areas, and poorest socio-economic statuses (18). The findings that a higher occurrence of AF among boys compared to girls are also reported by the works of Fenta and others (2021) in Ethiopia (12). The studies by Khamis and others (2020) in Tanzania and Vollmer and others (2017) in Bangladesh also recorded a higher occurrence of AF among the non-or-less educated mothers than their counterparts (11, 41).

In conclusion, the higher prevalence of anthropometric failure in India embodies a major area of concern to study for getting a healthier society. The regional variation in the occurrence of anthropometric failure represents the influence of various factors in different areas. Some factors influence anthropometric failures uniformly across all regions, whereas others have regionally specific impacts. These aspects imply that regional determinants should be taken into considerations when implementing child nutrition development programs.

To reduce the demographic failure of Indian children, it is pressingly necessary to prioritize steps to reduce childhood anemia, improve maternal education, raise the wealth index per family, and take special care of children from Scheduled Tribes and Scheduled Castes. Understanding the taxing factors by the researchers will be helping the policymakers to plan and execute a required strategy to mitigate those problems.

India, a developing nation, has a very high prevalence of undernutrition in children under the age of five. The effects of undernutrition on the child nutrition are assessed by AF by combining three indices of child malnutrition, i.e. wasting, stunting, and underweight. In the current context, it appears more reasonable to include these indications together in AF in order to determine factors that are responsible. Although AF has been separately studied in various communities or regions, there hasn't been a regional distribution of the entire nation; for this reason, the regional distribution has been shown in this study. This study will aid governments and non-governmental organisations in the implementation of future nutritional development initiatives for children, allowing India to easily reach Sustainable Development Goals (SDGs) targets. As the present study is based on secondary data source, the analysis is limited to the available data set only. More primary studies in broader perspectives would be helpful to get vast information of Indian population regarding malnutrition.
